# Supplementary material for: From sequence to enzyme mechanism using multi-label machine learning
Source: BMC Bioinformatics. 2014 May 19;15:150. doi: 10.1186/1471-2105-15-150 (PMC4229970; doi:10.1186/1471-2105-15-150)
Supplement: Additional file 2 — Java code of ml2db. Additional file ml2db_code.tar.gz contains the Java source code to run the multi-label machine learning experiments and save the results to database. The code’s Javadoc is included. [file 1471-2105-15-150-S2.zip › additional file 2/ml2db/ml2db/doc/mulan/classifier/neural/model/ActivationFunction.html]

ActivationFunction


JavaScript is disabled on your browser.


- Overview
- Package
- Class
- Use
- Tree
- Deprecated
- Index
- Help

- Prev Class
- Next Class

- Frames
- No Frames

- All Classes

- Summary:
- Nested |
- Field |
- Constr |
- Method

- Detail:
- Field |
- Constr |
- Method


mulan.classifier.neural.model

## Class ActivationFunction

- java.lang.Object
- - mulan.classifier.neural.model.ActivationFunction

- All Implemented Interfaces:
  :   java.io.Serializable

  Direct Known Subclasses:
  :   ActivationLinear, ActivationTANH

  ---

    

  ```
  public abstract class ActivationFunction
  extends java.lang.Object
  implements java.io.Serializable
  ```

  Abstract base class for activation functions.
  The activation function is used in neural network to transform an input of
  each layer (neuron) and produce the output for next layer (neuron).
  Depending on learning algorithm, derivation of activation function might be necessary.

  Version:
  :   2012.02.27

  Author:
  :   Jozef Vilcek

  See Also:
  :   Serialized Form

- - ### Constructor Summary

    Constructors

    Constructor and Description

    `ActivationFunction()`
  - ### Method Summary

    Methods

    | Modifier and Type | Method and Description |
    | --- | --- |
    | `abstract double` | `activate(double input)` Computes an output value of the function for given input. |
    | `abstract double` | `derivative(double input)` Computes an output value of function derivation for given input. |
    | `abstract double` | `getMax()` Gets the maximum value the function can output. |
    | `abstract double` | `getMin()` Gets the minimum value the function can output. |

    - ### Methods inherited from class java.lang.Object

      `equals, getClass, hashCode, notify, notifyAll, toString, wait, wait, wait`

- - ### Constructor Detail


    - #### ActivationFunction

      ```
      public ActivationFunction()
      ```
  - ### Method Detail


    - #### activate

      ```
      public abstract double activate(double input)
      ```

      Computes an output value of the function for given input.

      Parameters:
      :   `input` - the input value to the function

      Returns:
      :   the output value


    - #### derivative

      ```
      public abstract double derivative(double input)
      ```

      Computes an output value of function derivation for given input.

      Parameters:
      :   `input` - the input value to the function

      Returns:
      :   the output value


    - #### getMax

      ```
      public abstract double getMax()
      ```

      Gets the maximum value the function can output.

      Returns:
      :   maximum value of the function


    - #### getMin

      ```
      public abstract double getMin()
      ```

      Gets the minimum value the function can output.

      Returns:
      :   minimum value of the function


- Overview
- Package
- Class
- Use
- Tree
- Deprecated
- Index
- Help

- Prev Class
- Next Class

- Frames
- No Frames

- All Classes

- Summary:
- Nested |
- Field |
- Constr |
- Method

- Detail:
- Field |
- Constr |
- Method
